# Computational Stimulation of the Basal Ganglia Neurons with Cost Effective Delayed Gaussian Waveforms

**DOI:** 10.3389/fncom.2017.00073

**Published:** 2017-08-08

**Authors:** Mohammad Daneshzand, Miad Faezipour, Buket D. Barkana

**Affiliations:** ^1^D-BEST Lab, Departments of Computer Science and Engineering and Biomedical Engineering, University of Bridgeport Bridgeport, CT, United States; ^2^Department of Electrical Engineering, University of Bridgeport Bridgeport, CT, United States

**Keywords:** deep brain stimulation, Parkinson's Disease, basal ganglia network, energy efficiency, neuronal activity, Gaussian waveform with delay, synchronization level

## Abstract

Deep brain stimulation (DBS) has compelling results in the desynchronization of the basal ganglia neuronal activities and thus, is used in treating the motor symptoms of Parkinson's disease (PD). Accurate definition of DBS waveform parameters could avert tissue or electrode damage, increase the neuronal activity and reduce energy cost which will prolong the battery life, hence avoiding device replacement surgeries. This study considers the use of a charge balanced Gaussian waveform pattern as a method to disrupt the firing patterns of neuronal cell activity. A computational model was created to simulate ganglia cells and their interactions with thalamic neurons. From the model, we investigated the effects of modified DBS pulse shapes and proposed a delay period between the cathodic and anodic parts of the charge balanced Gaussian waveform to desynchronize the firing patterns of the GPe and GPi cells. The results of the proposed Gaussian waveform with delay outperformed that of rectangular DBS waveforms used in *in-vivo* experiments. The Gaussian Delay Gaussian (GDG) waveforms achieved lower number of misses in eliciting action potential while having a lower amplitude and shorter length of delay compared to numerous different pulse shapes. The amount of energy consumed in the basal ganglia network due to GDG waveforms was dropped by 22% in comparison with charge balanced Gaussian waveforms without any delay between the cathodic and anodic parts and was also 60% lower than a rectangular charged balanced pulse with a delay between the cathodic and anodic parts of the waveform. Furthermore, by defining a Synchronization Level metric, we observed that the GDG waveform was able to reduce the synchronization of GPi neurons more effectively than any other waveform. The promising results of GDG waveforms in terms of eliciting action potential, desynchronization of the basal ganglia neurons and reduction of energy consumption can potentially enhance the performance of DBS devices.

## Introduction

Parkinson's disease is associated with a complex variation in neuronal spiking patterns in the basal ganglia such as more observed burst and oscillatory patterns (Levy et al., 2000). Experimental recordings of patients with Parkinson's disease (PD) show an increase in burst firing patterns and synchronization in the Sub Thalamic Nucleus (STN) and Globus Pallidus inturnus (GPi) while a decrease in the firing rate of Globus Pallidus externus (GPe; Bergman et al., 1994; Nini et al., 1995; Wichmann et al., 1999). This synchronization happening in the beta range might be a source to intensify motor symptoms of PD (Brown, 2007; Rivlin-Etzion et al., 2010). In order to explain the original oscillation in PD, two hypotheses have been proposed in retrospective studies. The first one is based on *in-vitro* recordings of the STN-GPe network and suggests that low frequency oscillation is due to the reciprocally connected STN-GPe network behaving as a pacemaker (Reck et al., 2009). The second hypothesis claims that the abnormal correlated burst firing pattern is due to dopamine depletion of presynaptic neurons, which increases the effect of GABAergic transmission from GPe to STN (Baufreton and Mark, 2008). Researchers suggest that irregular firing in the basal ganglia neurons, characterizing neurological disorder such as PD, may arise locally and then propagate throughout the brain. But the underlying mechanism remains unclear. Tang et al. (2016) constructed a minimal neuron-astrocyte network model by connecting a neurons chain and an astrocytes chain to investigate the local propagation of abnormal firings.

Neuromodulation is a fast growing field of study focusing on methods of interfering with neuronal activity. Electrical stimulation such as Deep Brain Stimulation (DBS) influences a wide variety of mechanisms at neuronal and system level (Deniau et al., 2010). Parameter configuration of DBS differs from patient to patient and requires experimental and computational models for obtaining a decent efficiency (St George et al., 2010). DBS disrupts the oscillatory activity of cells within the basal ganglia (Tass, 2003; Modolo et al., 2008). The previous hypotheses about the mechanism of DBS were based on the idea of regularizing pathological activity through entrainment and synaptic modifications (Rubin and Terman, 2004; Dorval et al., 2010), while recent studies elucidated that DBS on STN causes complex changes in the firing rate of efferent structures (Humphries and Gurney, 2012).

Experimental studies investigated the effect of DBS on the single cell level (Hashimoto et al., 2003), but there are vast unknown information that can be extracted from neural network activity and its reaction to stimulation. There are several existing neural mass models aiming to understand ambiguities of DBS in the network level (So et al., 2012; Summerson et al., 2015). Terman et al. (2002) focused on synaptic interaction between STN and GPe to describe and compare normal and PD conditions. Along all the computational models used in retrospective studies, we used a modified version of the basal ganglia network (So et al., 2012) to investigate the effect of DBS parameters.

The neuronal electrical activities depend on a periodical force current or high frequency periodic DBS signals (Lv and Ma, 2016; Lv et al., 2016). High frequency DBS (more than 100 Hz) has more therapeutic effect than low frequency stimulations, and this effect would increase if pulses are given at a specific phase (McConnell et al., 2012). Efficient stimulus waveforms must be able to elicit action potentials that subsequently lead to the release of neurotransmitters while minimizing the side effects such as tissue damage, charge injection decrease, and increase in energy consumption (Lilly et al., 1955; Sahin and Tie, 2007; Jezernik et al., 2010). Recent stimulators have used charge balanced waveforms with short duration, high amplitude followed by long duration, low amplitude pulses. One of the optimal DBS waveforms which decreases energy consumption is an exponentially growing pulse by Jezernik and Manfred (2005). Wongsarnpigoon and Grill (2010) found a Gaussian waveform using the genetic algorithm to be the most optimal waveform. Most of the implanted stimulators generate a high frequency pulse train (Coffey, 2009). In order to have a charge balanced efficient stimulus pulse for PD, these pulses should have the amplitude around 3 V and a frequency of 130 Hz (Moro et al., 2002). A prolonged delay between the two parts of the charge balanced stimulation pulse improves activation of the resting neurons while entrainment of the bursting neurons (Hofmann et al., 2011).

In this work, we investigated the effect of this delay on more energy efficient waveforms in a biologically detailed network of the basal ganglia neurons. Optimized waveforms could prolong battery life, reduce the frequency of recharge intervals and reduce the cost and risk of battery replacement surgeries. The main contributions of this research can be listed below.

1. A combinational DBS waveform with a delay between the anodic and cathodic phases is proposed.
2. This modification of the DBS waveform allows for less energy consumption by the stimulus pulse train along with the reduction of the number of times that DBS waveform fails to elicit an action potential.
3. Experimental results show that this new DBS waveform can activate the basal ganglia neurons with lower amplitude and shorter duration of delay compared to previously used DBS signals such as rectangular pulses.
4. This new waveform modification also improves the synchrony of DBS pulses with firing of the basal ganglia neurons; i.e., it was able to elicit an action potential on the applied neuron with almost every pulse of the DBS signal.
5. Thorough analytic study of the desynchronization of the basal ganglia cells under PD condition has been carried out.

## Materials and methods

With the aid of differential equations, mathematical cell models provide an insight through synaptic and injected currents such as DBS and also regarding how these external currents affect the membrane voltage of cells. In addition, these models can be used to test the effects of DBS waveforms on neuronal firing patterns. Table 1 shows some of the most comprehensive retrospective studies. Some of these works focused on achieving a biological compatible neuronal model while others tested the effect of DBS waveforms along with the model structure. The model used in this research is a slightly modified version of the basal ganglia network proposed by So et al. (2012) due to its ability to consider the contribution of local cells in the basal ganglia under DBS currents.

**Table 1:** Previous works.

**Works**	**Methodology**	**Advantages**	**Disadvantages cons**
Rubin and Terman, 2004	Original RT model based on Hudgkin–Huxley equations.	Able to reproduce both pathological and physiological activities of STN, GPe, GPi, and Thalamic cells.	Does not consider the effect of sensory motor cortex excitatory inputs.
Pirini et al., 2009	Enhanced Rubin and Terman (RT) model with the effect of Striatum cells on the network and Rectangular DBS on different neuronal targets.	Representation of direct pathways of the basal ganglia cells.	Some phenomena like electrode to neuron distance, effects of somas, dendrites and axons, synaptic activation/inactivation effects, and neurotransmitter depletion are not considered by this work.
Foutz and McIntyre, 2010	Energy efficient non-rectangular DBS waveform.	Examining the neuronal activation energy in both intracellular and extracellular stimulation.	Proposed waveforms are hampered by increased charge requirements, which may limit potential savings in battery life.
Wongsarnpigoon and Grill, 2010	Guassian energy efficient DBS waveform with Genetic Algorithm.	Ability to find the optimal DBS waveform parameters with the Genetic Algorithm.	Lack of DBS targeting consideration.
Hofmann et al., 2011	Charge balanced DBS waveforms with introduction of a gap between cathodic and anodic phases.	Modified DBS waveforms showed a considerably increased efficiency in terms of activation and entrainments of neuronal activities.	Considering these DBS waveforms on a single compartment model rather than neuronal population network. Only considers rectangular pulses.
So et al., 2012	Enhanced RT model with rectangular DBS waveforms.	High ability to reconstruct the biological phenomena happening in the basal ganglia cells.	The model does not consider 3 dimensional orientations of different nuclei and the position of the stimulating electrode.
Summerson et al., 2015	Various charge balanced DBS waveforms implemented on a new cortical model.	High complexity and accuracy of model with considering the layer V into the model.	Irregular DBS waveform used does not provide the mean to understand the effect of DBS waveforms parameters.
Holt et al., 2016	Closed loop approach of Deep Brain Stimulation on the HM (Hahn and McIntyre, 2010) model.	Considers the closed loop phasic stimulation which enables applying the DBS waveform at a proper time.	The complexity of neuronal network is not fully addressed by the model.

### Basal ganglia network model

This model adopts the basic differential equations of cells by Hodgkin and Huxley (1952) in an interconnected manner to stimulate involving neurons in the basal ganglia such as STN, GPe, GPi, and Thalamic (Th) neurons. In our modified model, synaptic currents between neurons are showed in the form of the equation below:

(1) $$\begin{array}{r} I_{ion}=\;g_{ion}m_{\infty }^{M}h_{\infty }^{N}\left(V-E_{ion}\right) \end{array}$$

where *g*_*ion*_ is the conductance variable of each ion to or from the cell and $m_{\infty }^{M}$ and $h_{\infty }^{N}$ are activation and inactivation functions varying slightly for each ion involved in the cell (Hodgkin and Huxley, 1952). *V* is the membrane potential and *E*_*ion*_ is the equilibrium potential of the ion. Each group of cells are represented by the modified Hudgkin Huxley model as explained in the following sections:

#### Thalamic neurons

Thalamic cells receive inhibitory inputs from GPi cells and respond to that with an induced firing rate. In order to generate the subthreshold charge and discharge of thalamic cells, a depolarizing current is applied to the resting potential of Thalamic cells. The membrane potential of Thalamic cells are defined by this equation based on So et al. (2012).

(2) $$\begin{array}{r} C_{Th}\frac{dV}{dt}=\;-I_{L}-I_{Na}-I_{K}-I_{T}-I_{GPi\to Th}+I_{SMC} \end{array}$$

where the conductance values for Leaky, Sodium, Potassium and T-type low threshold spiking currents are 0.05, 3, 5, and 5 $\frac{mS}{cm^{2}}$ and the equilibrium potentials are −70, 50, −75, and 0 mV, respectively. *I*_*SMC*_ is the sensory motor cortex current representing the effect of other cells in the cortex on Thalamic neurons. *I*_*SMC*_ is defined as the normal distributed pulse train with a frequency of 14 Hz and coefficient variance of 0.2 with each pulse having the amplitude of 3 $\frac{\mu A}{cm^{2}}$ and duration of 5 ms, in order to generate the unordinary signal train of the motor cortex. *I*_*GPi*→*Th*_ represents the inhibitory currents from a GPi neuron to each Thalamic neuron with the conductance of 0.17 $\frac{mS}{cm^{2}}$ and equilibrium potential of −85 mV.

#### STN neurons

STN neurons receive many ionic currents including inhibitory projections from GPe cells, DBS currents and a constant bias current *I*_*bias*_, which is the accumulated synaptic current from other brain regions. STN neurons have low rate of firing without an external stimulus (Bevan and Wilson, 1999; So et al., 2012). The equation governing the membrane potential of STN cells is as follows.

(3) $$\begin{array}{r} C_{STN}\frac{dV}{dt}=\;-I_{L}-I_{Na}-I_{K}-I_{ca}{-I_{T-}I}_{GPe\to STN}\\ +I_{DBS}+I_{bias} \end{array}$$

where the conductance values for Leaky, Sodium, Potassium, Calcium, and T-type low threshold spiking currents are 2.25, 37, 45, 2, and 0.5 $\frac{mS}{cm^{2}}$ and the equilibrium potentials are −60, 55, −80, 140, and 0 mV, respectively. *I*_*GPe*→*STN*_ represents the inhibitory currents from 2 GPe neurons to each STN neuron with the conductance of 0.5 $\frac{mS}{cm^{2}}$ and equilibrium potential of −85 mV. *I*_*bias*_ is set to 29 $\frac{\mu A}{cm^{2}}$ for the healthy case and 20 $\frac{\mu A}{cm^{2}}$ for the Parkinsonian case and finally, *I*_*DBS*_ is the deep brain stimulus used in the model.

#### GPe and GPi neurons

The firing activity of Globus Pallidus Pars neurons increases with an increased stimulus signal and follows a continuous monotonic pattern (Kita and Kita, 2011; So et al., 2012). Equations (4) and (5) represent the GPe and GPi neurons in this computational model.

(4) $$\begin{array}{r} C_{GPE}\frac{dV}{dt}=\;-I_{L}-I_{Na}-I_{K}-I_{ca}{-I_{T-}I}_{STN\to GPe}{+I}_{GPe\to GPe}\\ +\;I_{DBS}+I_{bias} \end{array}$$

(5) $$\begin{array}{r} C_{GPi}\frac{dV}{dt}=\;-I_{L}-I_{Na}-I_{K}-I_{ca}{-I_{T-}I}_{STN\to GPi}{+I}_{GPe\to GPi}\\ +\;I_{DBS}+I_{bias} \end{array}$$

where the conductance values for Leaky, Sodium, Potassium, Calcium and T-type low threshold spiking currents are 0.1, 120, 30, 0.15, and 0.5 $\frac{mS}{cm^{2}}$ and the equilibrium potentials are −65, 55, −80, 120, and 0 mV, respectively and these values are the same for GPe and GPi neurons. *I*_*STN*→*GPe*_ represents the excitatory currents from two STN neurons to each GPe neuron with the conductance of 0.15 $\frac{mS}{cm^{2}}$ and equilibrium potential of 0 mV. *I*_*GPe*→*GPe*_ represents the inhibitory currents from two GPe neurons to each GPe neuron with the conductance of 0. 5 $\frac{mS}{cm^{2}}$ and equilibrium potential of −85 mV. *I*_*STN*→*GPi*_ represents the excitatory currents from two STN neurons to each GPi neuron with the conductance of 0.15 $\frac{mS}{cm^{2}}$ and equilibrium potential of 0 mV. *I*_*GPe*→*GPe*_ shows the inhibitory currents from two GPe neurons to each GPi neuron with the conductance of 0.5 $\frac{mS}{cm^{2}}$ and equilibrium potential of −85 mV. *I*_*bias*_ is set to 20 $\frac{\mu A}{cm^{2}}$ for the healthy case and 8 $\frac{\mu A}{cm^{2}}$ for the Parkinsonian case in Equation (4), 22 $\frac{\mu A}{cm^{2}}$ for the healthy case and 12 $\frac{\mu A}{cm^{2}}$ for the Parkinsonian case in Equation (5).

### Basal ganglia network and synaptic connections

The basal ganglia network proposed by So et al. (2012) claimed that running the model with 100 cells in each population would cause a small alteration of results in comparison with a smaller network with 16 cells in each population. Since a rectangular pulse shape similar to DBS was applied, increasing the network size will influence the DBS waveform capability. In our model, we set TH, STN, GPe and GPi populations, each with 1,000 neurons. The connection between these populations arises in conformity with the topological structure of the connectivity within the basal ganglia and thalamic cells (Smith et al., 1998). We assumed the synaptic connectivity between each population of neurons to be in the form of Equation (1) and in this regard, the number of connections from each type of neuron to other types is shown in Table 2.

**Table 2:** Number of connections between neuronal populations.

**From population *i***	**To population *j***	**Synaptic type**
2 STN	1 GPE	Excitatory
2 GPe	1 GPe	Inhibitory
2 STN	1 GPi	Excitatory
2 GPe	1 GPi	Inhibitory
2 GPe	1 STN	Inhibitory
1 GPi	1 Th	Inhibitory

*Number of excitatory/inhibitory synapsis between populations in the basal ganglia model. The first column indicates the number of presynaptic neurons connecting to a postsynaptic neuron in the second column*.

**Figure 1:**
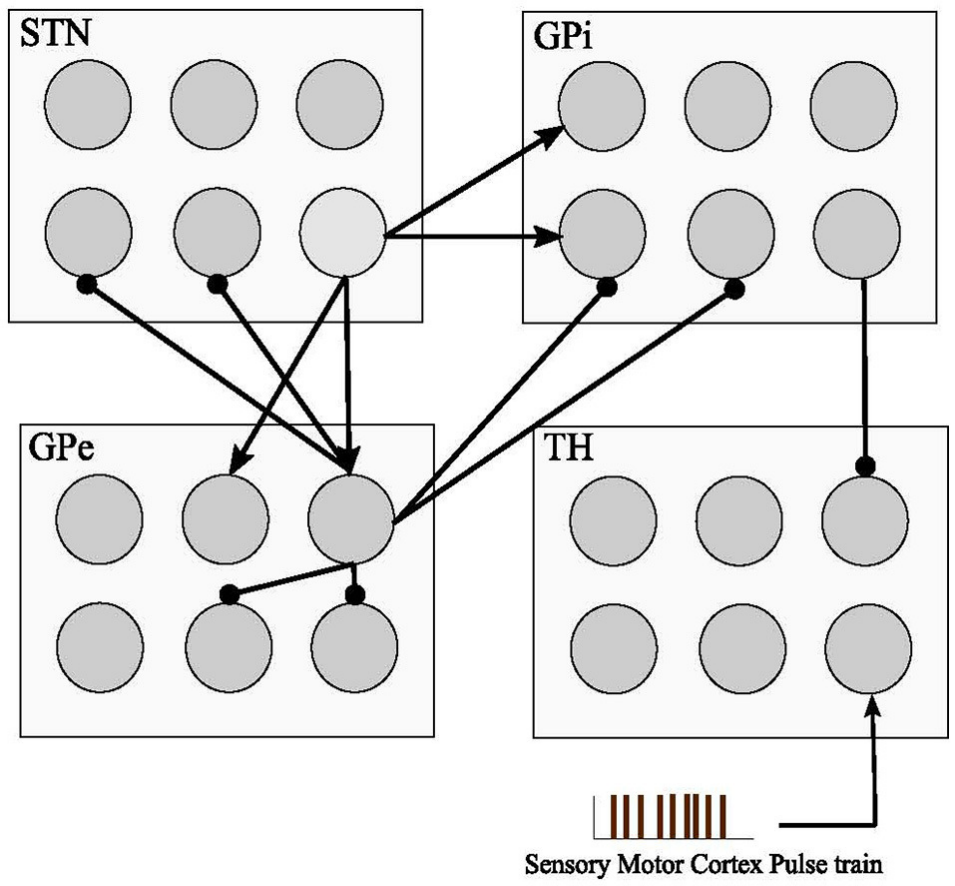
Basal Ganglia connectivity model. The model consists of four cell types with 1,000 single compartment model neurons in each type. Inhibitory connections are shown with (•) and excitatory inputs are shown with (▴). The sensory motor cortex pulse train is defined as an excitatory input to the thalamic cells from the accumulation of firing cells in the sensory motor cortex. STN, GPe, and GPi neuronal pools are subjected to Deep Brain Stimulation (DBS) in this model.

Each GPe cell has inhibitory projections to two STN, GPi, and GPe neurons while each STN neuron has two excitatory connections to two GPe and two GPi neurons, as shown in Figure 1. GPi neurons act as an output connection to Thalamic neurons with one inhibitory connection. This model also considers an irregular frequency pulse train as an input to the thalamic cells, exemplar of the accumulated current from the sensory motor cortex region. The pulse train has an amplitude of 3 $\frac{\mu A}{cm^{2}}$ and duration of 5 ms. The basal ganglia network by So et al. (2012) also considers a network current which is viewed as the input current *I*_*bias*_ from other brain regions to STN, GPe, and GPi cells which plays a significant role in the computational model to determine the healthy or Parkinsonian response of the network. By reducing this current going to STN, GPe, and GPi cells, an elevated bursting pattern and synchronization of GPe/GPi cells would emerge. Examination of deep brain signals were further achieved by applying customized pulse shapes to STN, GPe, and GPi cells, and compared with the typical rectangular waveform used in retrospective studies (Hofmann et al., 2011; So et al., 2012; Kang and Lowery, 2013; Summerson et al., 2015). It's been studied recently that fluctuations of neuronal action potentials generate a magnetic field potential. The effect of any external force such as electromagnetic radiation could be described by an input current in the neuronal loop (Lv and Ma, 2016; Lv et al., 2016). The DBS currents in our work induces the firing of a group of the basal ganglia neurons by generating repetitive (loop like) pulses. Generally, the effect of any external force such as electromagnetic radiation or DBS, could be modeled by an additive transmembrane current. Lv and Ma (2016) and Lv et al. (2016) proposed a dimensionless Hindmarsh-Rose model, considering magnetic flux in their model. Their model is greatly reliable for bifurcation analysis while being capable of reproducing chaotic firing activities within some neurons.

### DBS waveforms

Many studies have attempted to examine the therapeutic effects of DBS waveforms on patients with Parkinson's disease (Berney et al., 2002; Hashimoto et al., 2003; Rizzone et al., 2014) along with more computationally based studies such as Wongsarnpigoon and Grill (2010), Foutz and McIntyre (2010), So et al. (2012), Summerson et al. (2015), Kang and Lowery (2013), and Hofmann et al. (2011). However, the effect of DBS pulse modification with a delay in a complex network of the basal ganglia has not been fully investigated. A typical waveform to be used as DBS consists of a Cathodal pulse followed by a longer extent of an Anodal pulse (Coffey, 2009).

In order to achieve a charge balanced Gaussian waveform we must carefully define the width ratio between the cathodic and anodic pulse, which in our study, we established as 1:3.3 to guarantee enough time for the depolarization of the membrane potential to have the maximum efficiency of DBS. We compared a group of waveforms with a delay between the cathodic and anodic phases in the computational network of the basal ganglia to see how the DBS signals can activate the resting neurons along with the reduction of neuronal synchronization. These signals are rectangular pulses, sinusoid, and Gaussian along with three biphasic waveforms with a delay between the cathodic and anodic parts, which we call Pulse Delay Pulse (PDP), Sinusoid Delay Sinusoid (SDS), and Gaussian Delay Gaussian (GDG) waveforms. All these waveforms were able to decrease the synchronization of GPe and GPi cells with a cathodic amplitude of 200 μ*A*. The pulse duration of the cathodic phases were 0.3 ms which were enough to elicit an action potential with minimum energy consumption (Wongsarnpigoon and Grill, 2010). The anodic phase of the amplitude was −20 μ*A* and a duration of 1 ms was assigned. The delay of 0.7 ms showed a promising value for the activation of resting neurons in our basal ganglia network. These values were used to be consistent with previous studies (Foutz and McIntyre, 2010; Wongsarnpigoon and Grill, 2010; So et al., 2012; Summerson et al., 2015) and to have a better comparison of energy consumption for each waveform, but as we show further in this study, the cathodic and anodic amplitudes along with the delay length can be varied to reach the optimal DBS waveforms.

**Figure 2:**
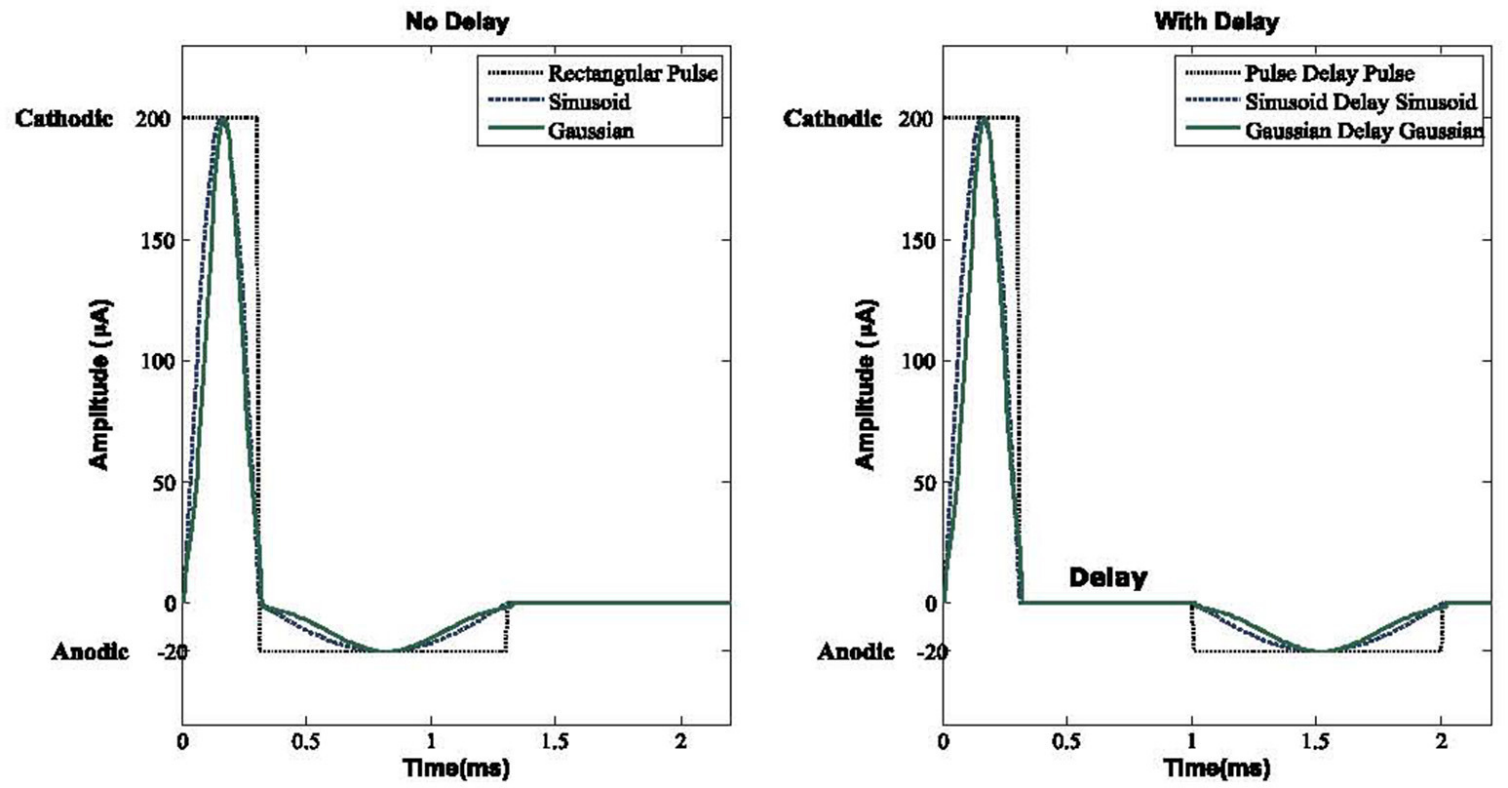
DBS waveforms. Three biphasic pulses with and without delay between the anodic and cathodic phases are used in this basal ganglia model. A delay elevates the threshold for the activation of neurons while making the network more resistant to the anodic phase. For both with and without delay Sinusoid and Gaussian waveforms, energy consumption would be lower compared to rectangular pulse shapes. For all waveforms, the cathodic, delay, and anodic phases have amplitudes of 200, 0, and −20 μ*A* and a duration of 0.3, 0.7, and 1 ms, respectively.

Placing a delay between the cathodic and anodic phases had been studied by Hofmann et al. (2011) with only a rectangular pulse gap pulse waveform implemented on a simple Hodgkin-Huxley and a Morris-Lacar model (Hodgkin and Huxley, 1952; Morris and Lecar, 1981), however it does not consider the interactive behavior of STN, GPe, and GPi neurons (Detorakis et al., 2015). They considered the effect of this DBS waveform by switching the cathodic and anodic phases, hence two waveforms of cathodic gap anodic (CGA) and anodic gap cathodic (AGC) were explored. They concluded that the AGC signals show a complex behavior in terms of threshold amplitude for action potential generation vs. gap duration. Therefore in this study, we defined our DBS signals in the form of a cathodic phase followed by a delay and then followed by an anodic phase. With this configuration, we were able to achieve the activation of resting neurons while having a fixed optimum delay length. The effect of a Gaussian delay Gaussian waveform has not been studied before which we showed is the most practical waveform for deep brain stimulation in terms of energy consumption, desynchronization of GPe/GPi cells, and performance of proper elicitation of action potential within the network. Figure 2 shows the waveforms we used in the computational basal ganglia model.

The anodic phase must have a lower amplitude with a longer duration to counter balance the effect of a short length but high amplitude cathodic pulse. With this configuration, we can minimize the tissue damage of patients going under DBS implantation (Harnack et al., 2004; DiLorenzo et al., 2014; Cogan et al., 2016). Interfering the DBS waveform with a delay between the cathodic and anodic parts also increases the threshold for the activation of neurons and will make the network less influenced by the anodic phase (Mortimer, 1990).

## Results

### Cost function

**Figure 3:**
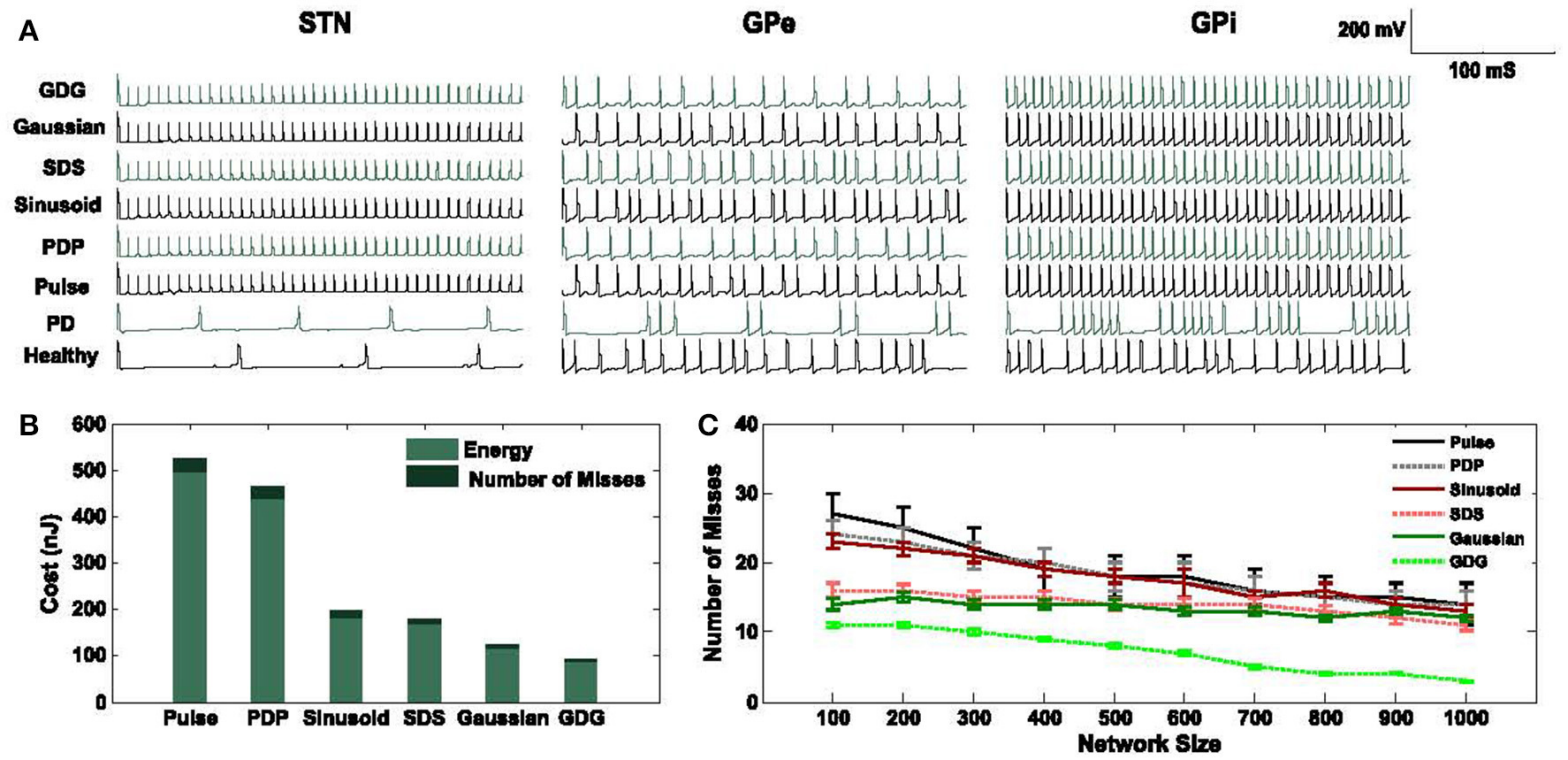
Cost evaluation of DBS waveforms. **(A)** STN, GPe, and GPi neuronal response to Pulse, Pulse Delay Pulse (PDP), Sinusoid, Sinusoid Delay Sinusoid (SDS), Gaussian and Gaussian Delay Gaussian (GDG) DBS waveforms plus Healthy, and Parkinsonian's Disease (PD) conditions. The network consists of 1,000 neurons in each cell type and the firing rates are averaged over multiple runs. **(B)** Total energy consumption of each DBS waveform of a network size of 1,000 neurons in each cell type. Waveforms with delays (PDP, SDS, and GDG) had less number of misses in eliciting an action potential while being relatively more energy efficient than the cathodic DBS waveforms. **(C)** The network size alters the accuracy of DBS waveforms in evoking action potentials while it is consistent with the energy consumption value. GDG waveforms show the lowest number of misses as the network size increases from 100 to 1,000 and they have a lower error on multiple runs of the network. Pulse, PDP, Sinusoid, and GDG waveforms are more sensitive to the network size while SDS and Gaussian waveforms reflect more steady responses in terms of the number of misses with network size variation.

We applied different pulse shapes as deep brain stimulators to investigate the energy consumed along with the number of times that the DBS signals failed to elicit an action potential. The cost function would simply accumulate these two criteria as the equation below:

(6) $$\begin{array}{r} C=\;\oint_{W}^{\;}{I\left(t\right)}^{2}Z\left(t\right)dt+M \end{array}$$

where *I*(*t*) is the DBS current waveform, *Z*(*t*) is the constant impedance set to 1 kΩ, *W* is the width of the waveform used and *M* is the number of misses in eliciting an action potential (each miss is considered with the penalty of 3 nJ). Each pulse of DBS tends to evoke an action potential on the implanted cell such as STN, GPe, or GPi. Ideally, the ratio of DBS pulse to neuron action potential is 1:1, but since this ratio in PD might not be exactly equal, variable *M* comes into picture. The width of waveforms is kept similar to each other to have a unified and fair comparison of cost functions. We use the basic pulse, Sinusoid and Gaussian (Wongsarnpigoon and Grill, 2010) waveforms and compare them to the modified waveforms with a delay between the cathodic and anodic phases, called Pulse delay Pulse (PDP), Sinusoid delay Sinusoid (SDS), and Gaussian Delay Gaussian (GDG). Figure 3 shows the DBS waveforms implemented in the computational network of the basal ganglia along with the amount of energy consumed by each waveform. As previously shown in Wongsarnpigoon and Grill (2010), Gaussian signals for DBS guarantee the minimum energy consumption compared to any other signal form such as pulse, rectangular, ramp, exponential, and sinusoid. Adding a delay followed by an anodic phase to the Gaussian waveform will slightly decrease the energy efficiency but provides lower error in eliciting action potentials. In Figure 3A, the neuronal responses of STN, GPe and GPi cells in the basal ganglia network are illustrated. There are two firing patterns for Healthy and Parkinsonian's Disease (PD) conditions along with the six types of DBS waveforms implemented. DBS waveforms were able to activate the basal ganglia neurons while suppressing the amplitude of spikes in STN neurons which is an output to GPi neurons (Hashimoto et al., 2003). STN neurons fired almost the same under all of the DBS waveforms, which shows that the stimulation of DBS neurons are independent of the DBS pulse shape. The lower amplitude of STN neurons is similar to the physical observation in Hollerman et al. (1992).

Synchronized responses of GPe and GPi neurons observed under PD condition were eliminated by all DBS waveforms although Gaussian and GDG waveforms were able to achieve more precise desynchronization. Beurrier et al. (1999) demonstrated that the increased firing rate of GPe and GPi neurons might be due to the depolarization of STN neurons. The network used in Figures 3A,B consists of 1,000 neurons in each cell type (Thalamic, STN, GPe, and GPi). The amount of energy consumed by GDG was almost one third of the rectangular pulse. Generally, waveforms with a delay between the cathodic and anodic parts (PDP, SDS, and GDG) showed less number of misses in eliciting an action potential every time they were implemented (Figure 3B). The results of energy consumption of these waveforms were almost consistent with the network size, but the number of misses in eliciting an action potential dropped by incrementing the network size as it went from 100 neurons in each cell type up to 1,000 (Figure 3C). We numerically evaluated the energy and number of misses on different network sizes to determine the consistency of the network. The error bars in Figure 3B state that through numerous runs of the network with different DBS waveforms, GDG showed the most persistent results.

### Firing patterns of basal ganglia neurons

**Figure 4:**
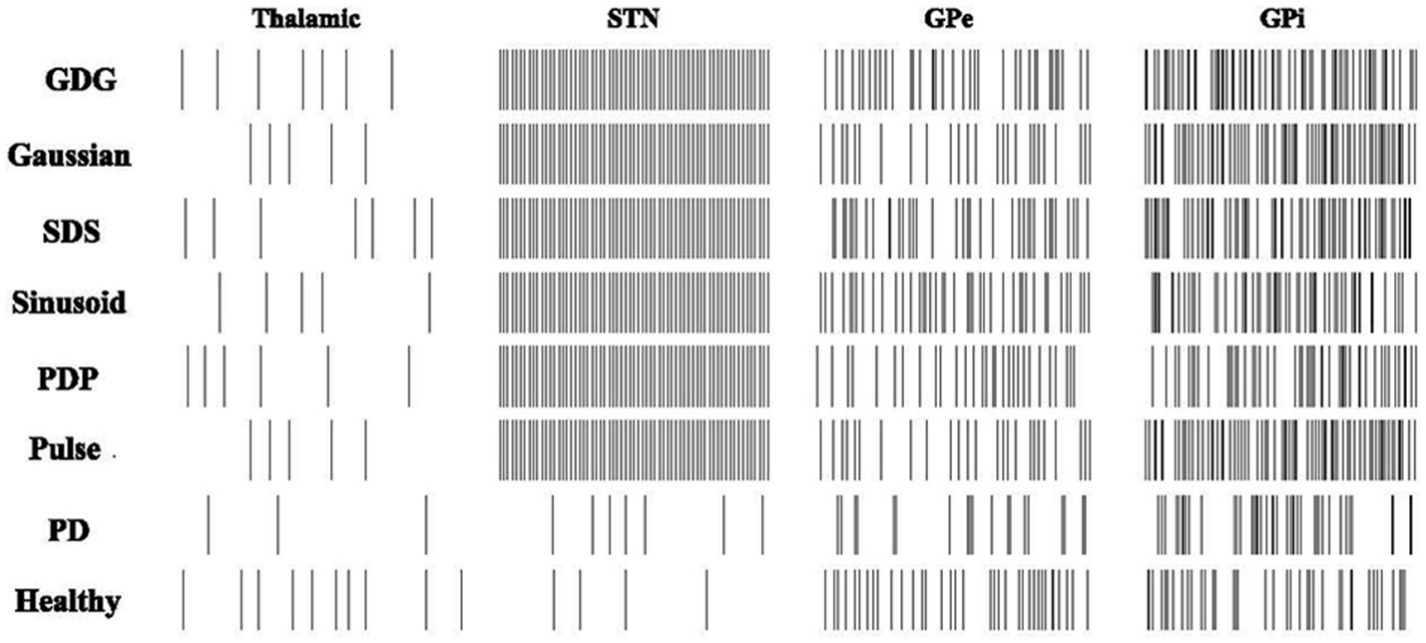
Firing patterns of the Basal Ganglia neurons. Raster changes of firing patterns from healthy and PD conditions to DBS implemented waveforms. PD decreases the firing rate of Thalamic, GPe and GPi neurons. Rectangular Pulses will adjust the neuronal response of Thalamic, GPe, and GPi neurons while failing to follow up with the STN firing in the Healthy condition. PDP, Sinusoid, SDS, Gaussian, and GDG will increase the firing rate of STN, GPe, and GPi cells compared to the PD condition and they also eradicate the synchronization of GPe and GPi neurons. GDG waveform results of GPe firing rates were promising as it simply adjusts itself to GPe firing pattern under Healthy conditions.

From the recording of the neuronal firing rates in monkeys (Bergman et al., 1994; Boraud et al., 1998; Wichmann and Soares, 2006), we expect to see an increase in the firing rate of GPi and STN neurons after implementing the DBS signals, while GPe neurons show the reduced firing rate. In Figure 4, the mean firing rate of STN, GPe, and GPi cells increased from the PD condition to the DBS implemented condition. From Healthy to PD condition, a decrease in the rate of GPe neurons is observable which is consistent with the recording data (So et al., 2012). All six DBS waveform types successfully increased the rate of spiking in Thalamic, STN, GPe, and GPi cells, however the amount of this boost in neuronal firing was lower for GPe neurons under the stimulation of a GDG waveform, which promises better results in diminishing the effects of Parkinson's disease (Wichmann et al., 1994). The relative increase in firing rates of GPe and GPi cells from PD to DBS implemented conditions corresponded to the experimental recordings in which the GDG and PDG waveforms in our model reached the highest comparability to the actual data (Hashimoto et al., 2003). The firing rate of Thalamic neurons decreases from healthy to PD condition while it increases after applying DBS, adjusted to Healthy conditions. Summerson et al. (2015) investigated the average firing rate of cells in the basal ganglia under regular and irregular rectangular pulse DBS waveforms. The rate did not differ noticeably in both cases proving that DBS has a uniform effect on the firing rate of single neurons, but with various waveform shapes and the effect of Delay between cathodic and anodic phases, GPi neurons tend to fire more. This increased firing pattern is due to counteracting the regularization period (Delay plus the anodic phase) and is essential to improve the outgoing signals from the basal ganglia to the thalamic neurons (Humphries and Gurney, 2012).

Synchronization of brain waves is the basis of functional connectivity for neural decoding (Fell and Axmacher, 2011). Changes in synchronization causes brain disorders like PD (Plenz and Kital, 1999; Moazami-Goudarzi et al., 2008). There are several bivariate techniques to study the synchronization in firing patterns of brain cells (Jalili et al., 2014), namely, the mutual information, phase locking (Guevara et al., 2005; Sakkalis et al., 2009; Rummel et al., 2011) and synchronization levels based on the correlation coefficient (Pirini et al., 2009). STN, GPe, and GPi neurons fire with various patterns under different DBS waveforms, therefore the similarity and synchronization of these firing responses compared to the firing patterns of these neurons under healthy condition is investigated by the bivariate techniques mentioned above. Based on Lv and Ma (2016) and Lv et al. (2016), appropriate external radiation can change the initial bursting of neurons into a switching mode of tonic and burst firing. This shows a promising finding in PD since many neurons tend to fire in switching mode due to PD. Also, in our work, the basal ganglia neurons under DBS showed numerous firing patterns and low DBS frequency signals were observed to fluctuate the neuronal activity into more switching patterns. Ma et al. (2017) stated that bursting synchronization under flux coupling could enhance with increasing external force current. Here, we also showed that DBS waveforms could affect the firing of population of neurons. For the STN neurons firing rate in Figure 4, we can see that once the amplitude of the DBS current is sufficient, the regular bursting emerges.

#### Mutual information

**Figure 5:**
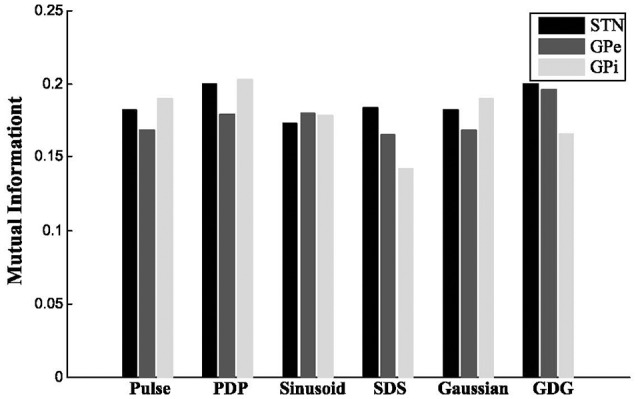
Mutual information. For STN and GPe neurons, the firing patterns under GDG waveforms (*Y*_*GDG*_) had the most amount of MI with *Y*_*H*_. Also for STN neurons, DBS waveforms with delay (*Y*_*PDP*_, *Y*_*SDS*_, and *Y*_*GDG*_) had higher MI compared to normal DBS waveforms (*Y*_*Pulse*_, *Y*_*Sinusoid*_, *Y*_*Gaussian*_). *Y*_*PDP*_ had more MI with *Y*_*H*_ than normal pulses for all neurons. *Y*_*SDS*_ for GPe and GPi shared lower MI with *Y*_*H*_ for both STN and GPe, while having lower MI for GPi cells.

Based on the correlation and coherence definitions, it seems that the relation between *Y*_*H*_ and *Y*_*DBS*_ of the basal ganglia neurons has non-linear characteristics, hence the mutual information can be more useful. The Mutual Information (MI) between two firing patterns is defined as below:

(7) $$\begin{array}{r} MI\left(Y_{H},Y_{DBS}\right)=\;\sum_{i\;=\;1}^{M}\sum_{j\;=\;1}^{M}P_{Y_{H}Y_{DBS}}^{ij}\ln\left(\frac{P_{Y_{H}Y_{DBS}}^{ij}}{P_{Y_{H}}^{i}P_{Y_{DBS}}^{j}}\right) \end{array}$$

where $P_{Y_{H}Y_{DBS}}^{ij}$is the estimated joint probability of the outcomes *i* and *j* for the signals and $P_{Y_{H}}^{i}$ is the estimated probability distribution of the *i*th outcome of *Y*_*H*_. For STN and GPe neurons, the firing patterns under GDG waveforms (*Y*_*GDG*_) had the most amount of MI with *Y*_*H*_. Moreover, for STN neurons, DBS waveforms with delay (*Y*_*PDP*_, *Y*_*SDS*_ and *Y*_*GDG*_), had higher MI compared to normal DBS waveforms (*Y*_*Pulse*_, *Y*_*Sinusoid*_, *Y*_*Gaussian*_). *Y*_*PDP*_ had more MI with *Y*_*H*_ than normal pulses for all neurons. *Y*_*SDS*_ for GPe and GPi shared lower MI with *Y*_*H*_ for both STN and GPe, while having lower MI for GPi cells (Figure 5).

#### Phase locking value

In order to understand the phase synchrony between DBS induced firing of STN, GPe, and GPi with the healthy condition, we used the Phase Locking Value (PLV; Aydore et al., 2013). First of all, we must extract the instantaneous phases from the signals using a Hilbert transform as below:

(8) $$\begin{array}{r} \varphi_{Y}={\text{tan}}^{-1}\;(\frac{H(Y)}{Y}) \end{array}$$

where *H*(*Y*) is the Hilbert transform of signal *Y*. In order to see how well the firing patterns of STN, GPe, and GPi neurons under DBS (*Y*_*DBS*_) is phase locked to the healthy condition (*Y*_*H*_), we can use the following measure:

(9) $$\begin{array}{r} \text{PLV}(Y_{H},Y_{DBS})=\;\frac{1}{L}\left|{\sum^{\text{​}}}_{i\;=\;1}^{L}e^{j\left(\varphi_{Y_{H}}-\varphi_{Y_{DBS}}\right)}\right| \end{array}$$

**Figure 6:**
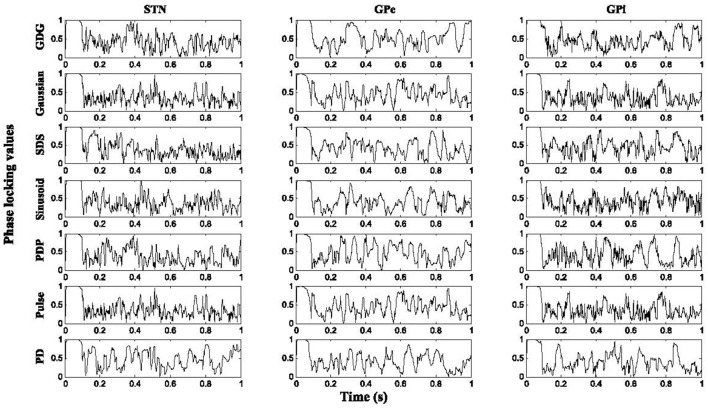
Phase locking values. Higher results of PLV were obtained with DBS waveforms modified with delay for all cell types. The firing pattern of all neurons was relatively more locked to healthy conditions under Gaussian and GDG waveforms.

Figure 6 shows the result of PLVs for STN, GPe, and GPi neurons under various DBS waveforms in comparison with *Y*_*H*_. For all neurons under DBS waveform with delay (*Y*_*PDP*_, *Y*_*SDS*_, and *Y*_*GDG*_), the PLV results were higher than firing under normal DBS (*Y*_*Pulse*_, *Y*_*Sinusoid*_, *Y*_*Gaussian*_), which shows that adding the delay in a charge balanced DBS waveform might provide more synchronized firing patterns to the healthy condition. The firing pattern of all neurons was relatively more locked to healthy conditions under Gaussian and GDG waveforms (*Y*_*Gaussian*_ *and Y*_*GDG*_). In PD condition, the PLV showed more tonic responses for all neurons which indicates the ability of the basal ganglia model to represent the irregular phases in which cells fire due to PD.

#### Synchronization level

**Figure 7:**
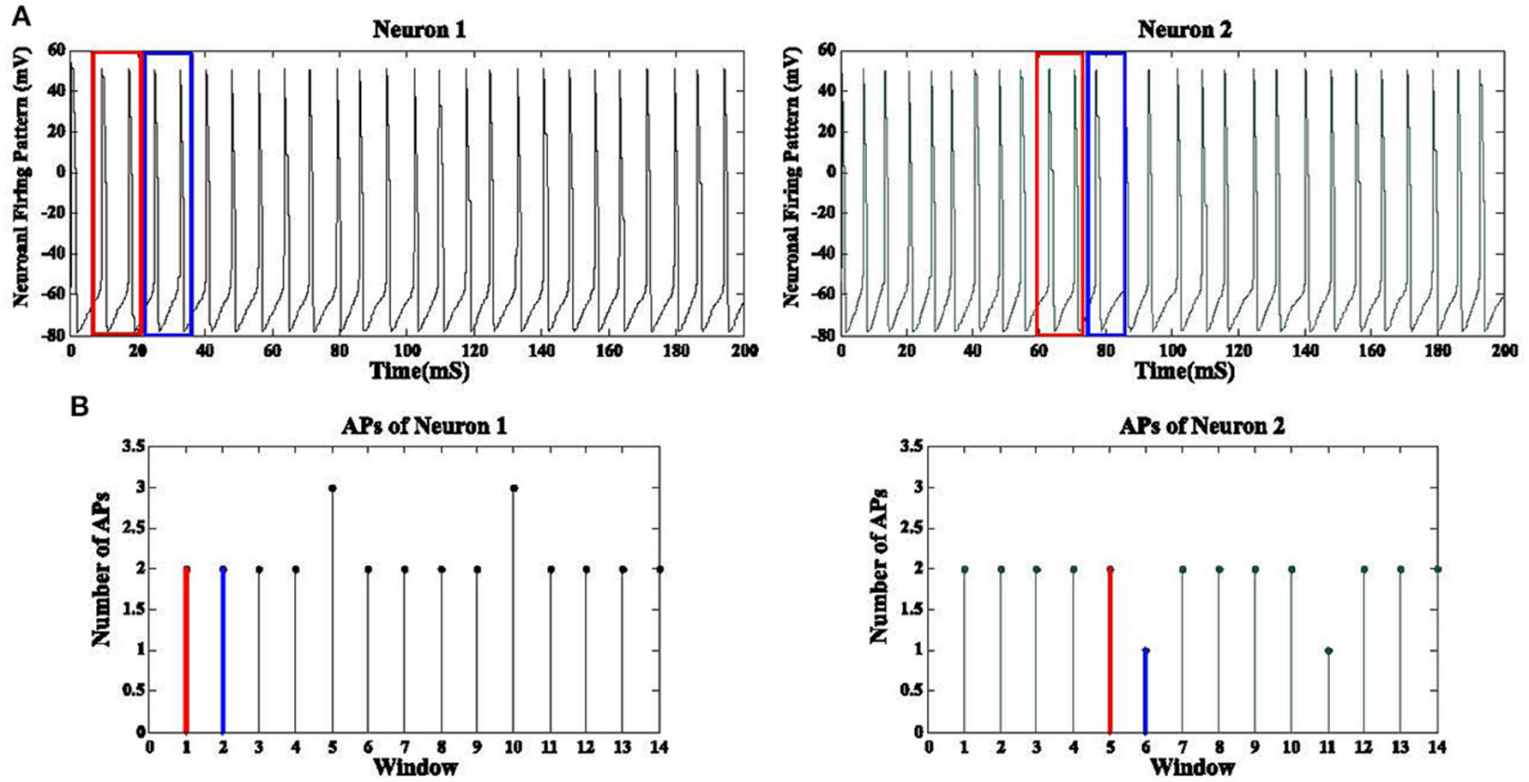
Synchronization level of GPi neurons. **(A)** Neuronal firing pattern of two GPi cells are shown for a duration of 200 mS. The blue and red boxes show the windows used to detect the APs inside them. Panel **(B)** shows the corresponding number of APs for each window. The number of APs for the red and blue boxes are shown in red and blue, respectively. The correlation coefficient between the discrete functions obtained from **(B)** is then calculated. A total of 1,000 GPi firing responses will produce 1,000 discrete functions as **(B)** and therefore a 1,000 by 1,000 correlation coefficient matrix is achieved for all GPi neurons.

GPi neurons tend to fire in a synchronized manner due to Parkinson's disease while DBS waveforms have a desynchronization effect on these firing patterns. The correlation between firing pattern of GPi neurons does not provide a meaningful metric to evaluate the level of synchronization due to extraneous low amplitude action potentials (AP) existing between the desired APs (Figure 7). Therefore, we applied a correlation coefficient analysis based on the desired APs of these neurons in order to measure the synchronization level among GPi neurons (Pirini et al., 2009). First of all, assuming a population of 1,000 GPi neurons, we extracted the number of action potentials for each non-overlapping frame (15 ms) for all GPi neurons. These APs were obtained by a threshold value of −20 mV. This would give us a function of the number of APs based on each frame for every GPi neuron (Figure 7). The reason for using non-overlapping windows is to avoid counting any AP more than once. Equation (10) shows the number of APs for neuron *i* in *k* consecutive frames:

(10) $$\begin{array}{r} F_{i}\left(j\right)=\;AP_{w\left(k\right)} \end{array}$$

*AP*_*w*(*k*)_ represents the number of action potentials calculated in the *k*th frame. In the next step, the correlation coefficient of these functions is defined for all GPi neurons. The correlation coefficient between 1,000 of functions *F_i_* is calculated in Equation (11).

(11) $$\begin{array}{r} \rho \left(F_{i},F_{j}\right)=\frac{\sum_{t\;=\;1}^{L}\left(F_{i}-mean\left(F_{i}\right)\right)\left(F_{j}-mean\left(F_{j}\right)\right)}{\sqrt{\sum_{t\;=\;1}^{L}{\left(F_{i}-mean\left(F_{i}\right)\right)}^{2}\sum_{t\;=\;1}^{L}{\left(F_{j}-mean\left(F_{j}\right)\right)}^{2}}} \end{array}$$

*L* is the number of elements in each function. For a GPi population of 1,000 neurons, a 1,000 by 1,000 elements correlation matrix *M*_*C*_ between all GPi neurons is generated using Equation (11).

The total number of values in matrix *M*_*C*_ with significant correlation (α ≤ 0.05) is called *N*_*S*_ while the total number of elements in *M*_*C*_ is denoted by *N*_*T*_. The synchronization level (SL) for the GPi neurons is finally derived by the equation below:

(12) $$\begin{array}{r} SL=\;\frac{N_{S}}{N_{T}} \end{array}$$

Table 3 shows the SL values of GPi neurons for different conditions. SL was calculated as 0.2 for the healthy condition. The low SL value shows that the synchronization of GPi neurons is low. The SL value was calculated as 0.733 for PD cases. This result shows an increased synchronization of GPi neurons. Higher synchronization due to PD was reported in Hammond et al. (2007) and Brown et al. (2004). However, it was not expressed quantitatively. The SL value proposed by this work allows for quantitative analysis to be performed. All DBS waveforms were able to reduce the SL of GPi neurons. The delay within the DBS signals (PDP, SDS, and GDG) reduced the SL values further, which shows that the delay in DBS signals could desynchronize the GPi firing patterns more effectively compared to normal DBS waveforms (including Rectangular Pulse, Sinusoid, and Gaussian). GDG waveforms had the lowest SL among all well-known DBS signal shapes. This states that Gaussian DBS waveforms are as efficient as rectangular pulses in term of desynchronization of GPi neurons. Also, charge balanced Gaussian waveforms with a delay between the cathodic and anodic phases can reduce the synchronization of GPi neurons due to PD even further in comparison with normal charge balanced DBS waveforms without delay.

**Table 3:** Synchronization level of GPi neurons.

**Condition**	**SL**
Healthy	0.2
PD	0.733
Rectangular Pulse	0.112
PDP	0.0889
Sinusoid	0.156
SDS	0.133
Gaussian	0.111
SDS	0.067

### Effect of delay on optimal DBS waveforms

**Figure 8:**
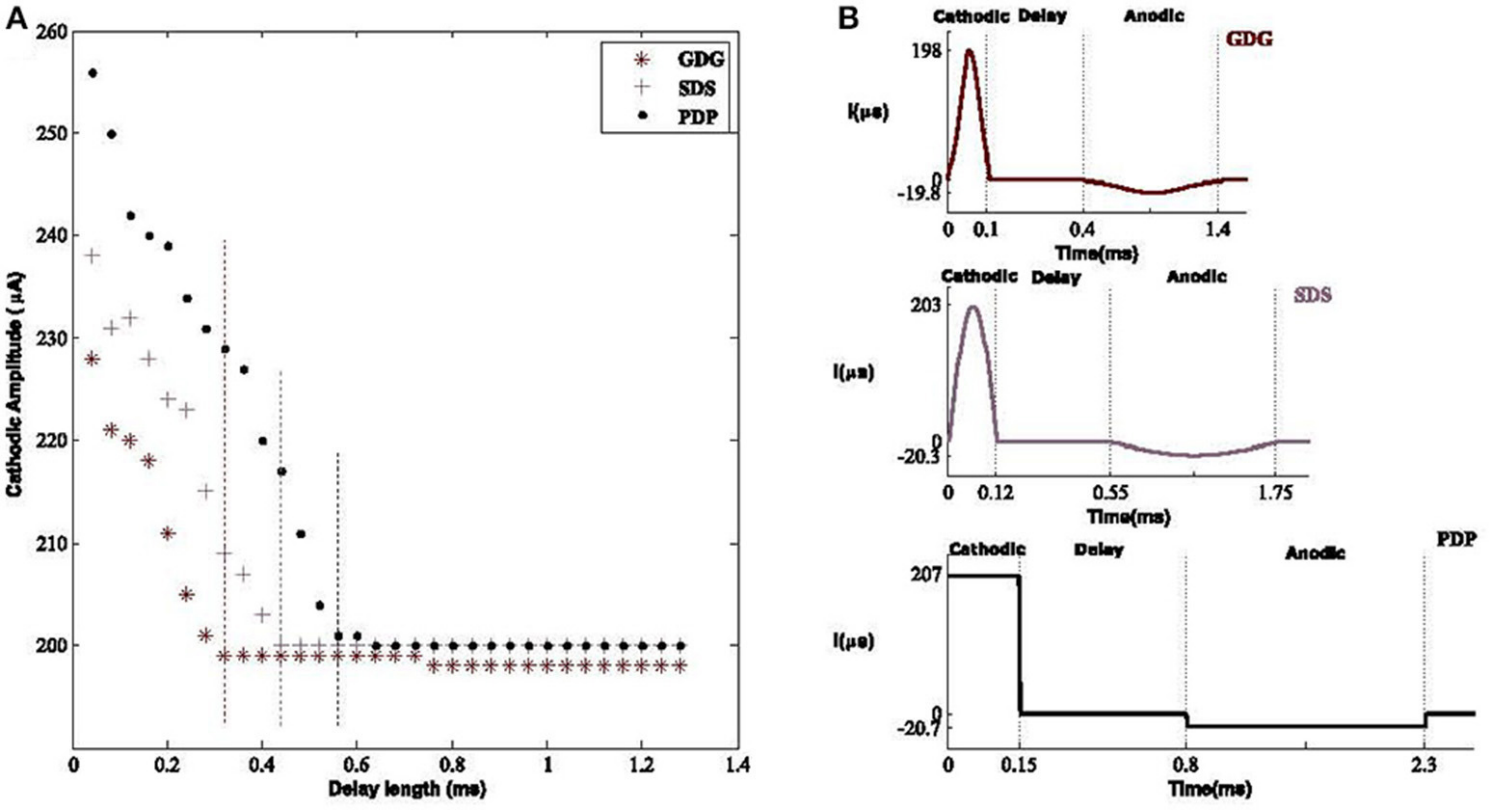
Optimal amplitude of DBS waveforms. **(A)** The effective cathodic amplitude to elicit an action potential for Gaussian Delay Gaussian (GDG), Sinusoid Delay Sinusoid (SDS), and rectangular Pulse Delay Pulse (PDP) DBS waveforms. The threshold of delay to reach the amplitude of 200 μ*A*, capable of eliciting action potentials was 0.32, 0.43, and 0.65 mS with respect to GDG, SDS, and PDP waveforms. Vertical dashed lines show the delay threshold values for each waveform. Delay sizes larger than these values lead to the same amplitude values and thus were unnecessary to plot. The GDG waveforms reached a lower delay threshold faster than SDS and PDP. **(B)** Optimal waveform shapes used in our basal ganglia network. The amplitude and duration ratio of the cathodic to anodic phases were 10:1 and 3.3:1, respectively.

The six DBS waveforms were examined for the capability of activating the resting neurons within the basal ganglia. Modification of delay length will provide a lower amplitude waveform in order to elicit an action potential. The main reason for this phenomenon is that the anodic phase will reverse the depolarization effect of the cathodic phase but with a delay in between, the cathodic phase has sufficient time to depolarize the membrane potential. Lower amplitude cathodic phases are able to depolarize the membrane potential but the process needs more time before the suppressive effect of the anodic phase (Hofmann et al., 2011), therefore a tolerable delay length is obligated to meet the desired condition. What we observed was that having a longer delay, reduces the amplitude of the DBS signal capable of eliciting AP's. Therefore, the total amount of energy is decreased. Although most of the energy consumption is related to the peak of the signal rather than its shape (see Equation 6; Wongsarnpigoon and Grill, 2010), showed that shape of the DBS pulse in population models, also contributes to this matter. This was also shown in our population model in which Gaussian signals with or without delay had lower effective amplitudes compared to other waveforms, as shown in Figure 8.

To study the delay effect in our basal ganglia network, we used three DBS waveforms with a variant delay length between the cathodic and anodic phases. The effect of delay length is seen on the waveform amplitude capable of eliciting an action potential. The Gaussian Delay Gaussian waveforms (GDG) were able to decrease the cathodic amplitude for eliciting an action potential because the cathodic Gaussian waveform will depolarize the membrane of the basal ganglia cells while the gradual decrease of the following anodic Gaussian phase will act as an additional delay for the depolarization process by creating a less sensitive waveform to the anodic phase (Figure 8A). The GDG waveforms reached the delay threshold faster than SDS and PDP. In this figure, the duration of the anodic phase was 3.3 times of the cathodic phase and the ratio of the cathodic to anodic phases is set to 10:1. The threshold of the delay to reach the amplitude of 200 μ*A*, capable of eliciting action potentials was 0.32, 0.43, and 0.65 mS with respect to GDG, SDS, and PDP waveforms. The obtained cathodic amplitude to elicit an action potential for PDP waveform was 30% lower than previous studies (So et al., 2012), while SDS and GDG had even lower amplitudes than PDP. The optimal values for these waveforms are shown in Figure 8B. GDG had the lowest cathodic amplitude while functioning with relatively lower amplitude in the anodic phase compared to the SDS waveform (−19 μ*A* for GDG anodic and −23 μ*A* for SDS). Lv et al. (2016) proves that the discharge mode of neuronal firing could be dominated by angular frequency when low amplitude is used in the external forcing current. Similar results in our work explain how introducing delay in the DBS current can reduce the amplitude of the external forcing current (DBS).

**Figure 9:**
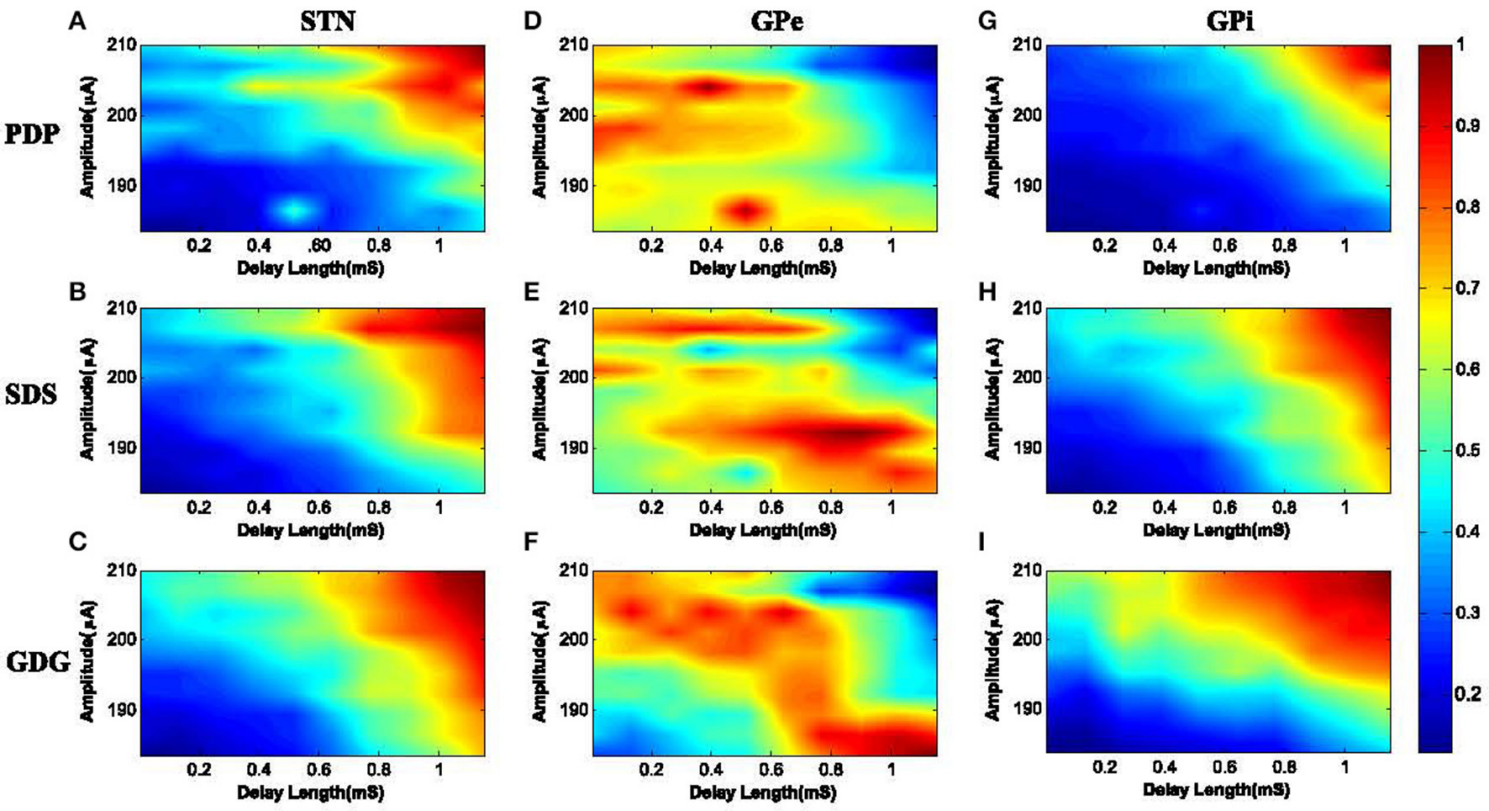
PLV of neuronal response with the DBS input. **(A)** STN neurons under PDP-DBS showed low PLV. Under SDS and GDG, higher PLV was obtained with shorter delay length and lower amplitude of the DBS signal, as seen in **(B,C)**. Under PDP, SDS and GDG waveforms, GPe neurons showed low synchronization and only for a short period of delay length, high synchronization was observed in **(D–F)**. The PLV results of GPi neurons with the input DBS were more consistent with STN neurons since they receive direct excitatory connections from STN. High synchronization for GPi neurons under PDP input happened at high amplitude and long delay lengths **(G)**. For SDS waveforms, to achieve high synchronization with GPi firing, the delay length and amplitude were a bit lower than PDP waveforms **(H)**. GDG signals outperformed PDP and SDS, as their high synchronization with GPi firings occurred at a much shorter delay length and lower amplitudes **(I)**.

It is necessary to evaluate the performance of DBS waveforms to see if the frequency of bursting neurons lock or synchronize to the DBS frequency. We investigated the capability of various pulse shapes to synchronize with the neuronal activity during simulation. We used the PLV metric to calculate the synchronization of bursting neurons with the input DBS waveform as explained by Equation (9). The 3 different DBS waveforms examined here were PDP, SDS and GDG. The results of synchronization (PLV) were obtained based on the delay length and the amplitude of the DBS signal. As shown in Figure 9, we increased the delay length from 0 to 1.2 mS with steps of 0.05 mS and also the amplitude of DBS signal was set to 190 μ*A* and increased with steps of 1 μ*A* up to 210 μ*A*. DBS frequency was set to 130 Hz and for each pair of delay length and amplitude, the PLV of the specified DBS waveform with the firing pattern of the desired neurons was obtained. We will discuss the effect of each DBS waveform shape on STN, GPe, and GPi neurons, but based on Figure 9, we can say that: for small amplitudes, the synchronization was low (PLV ≤ 0.2) meaning that neuronal firing was not adapted with the input DBS signal. Increasing the amplitude did not increase the synchronization for zero or small values of the delay length (PLV ≤ 0.5). Moreover, DBS waveforms with long delay but low amplitude, failed to achieve high synchronization (PLV ≤ 0.6). Once the amplitude and delay length were sufficiently increased, near optimal synchronization was observed (PLV ≥ 0.9). Furthermore, it has been shown that the anodic phase of the DBS waveform has a hyper polarization effect (Hofmann et al., 2011). Hyperpolarization helps enhancing the speed of relaxation of the neurons and faster relaxation implies a more bursting pattern. Increased bursting pattern of neurons advances the synchronization with the input DBS. The delay also provides enough relaxation time, hence higher delay length with sufficient amplitude followed by the anodic phase, leads to more synchronization of bursting neurons with the input stimuli.

The PLVs of STN burst firing with PDP, SDS, and GDG waveforms are shown in Figures 9A–C, respectively. Synchronization of STN neurons with PDP occurred at delay length and amplitude higher than 0.8 mS and 203 μ*A* (PLV ≥ 0.9). Under SDS input, synchronization occurred at a slightly smaller delay length and amplitude. The desired PLV of STN neurons with GDG input stimuli happened at delay length of 0.7 mS and amplitude of 192 μ*A*. The slow increasing slope of Gaussian anodic phase in GDG waveform acts as an extra delay, therefore, higher synchronization with lower delay length is achievable between GDG input and STN burst firing. For PDP, SDS, and GDG waveforms, GPe neurons showed low synchronization and only for a short period of delay length, high synchronization was observed (Figures 9D–F). Generally, synchronization of GPe burst firing with the input DBS signal was low due to the fact that the DBS signals are targeted to STN neurons in this model. Also, the inhibitory connections between GPe and STN neurons might have disruptive effect on the burst firing patterns of GPe neurons causing these neurons not to accommodate the DBS signal. The PLV results of GPi neurons with the input DBS were more consistent with STN neurons since they receive direct excitatory connections from STN (Figure 1). High synchronization (PLV ≥ 0.8) for GPi neurons under PDP input happened at a delay length more than 0.9 mS and amplitudes higher than 200 μ*A* (Figure 9G). For SDS waveforms, to achieve high synchronization with GPi firing (PLV ≥ 0.8), the delay length and amplitude must be higher than 0.8 mS and 200 μ*A*, respectively, as shown in Figure 9H. GDG signals outperformed PDP and SDS as their high synchronization with GPi firings (PLV ≥ 0.8) occurred at a delay length longer than 0.6 mS and amplitudes higher than 198 μ*A*.

We can summarize the synchronization effect of different DBS signal shapes with neuronal burst firing as follows:

- The anodic phase of DBS waveforms act as a hyper polarizer, causing reduction of neuronal relaxation time and therefore, speeding up the bursting frequency. Faster bursting leads to more synchronization of the DBS signal and neuronal firings.
- GDG waveforms were able to achieve high PLVs with neuronal burst firing while having a shorter delay length compared to PDP and SDS specifications. The slowly uprising anodic Gaussian phase of GDG waveforms compensate for the shorter delay length.
- High synchronization of GDG waveforms and neuronal burst firing (PLV ≥ 0.7) was obtained with a lower cathodic amplitude, which makes them more energy efficient compared to SDS or PDP waveforms.
- As shown in Figure 9, if the delay length is long enough, all neuron types under any DBS waveforms show very high synchronization (PLV ≥ 0.9). This shows the high effectiveness achieved by adding the delay between the cathodic and anodic phases of the DBS signal.
- Increasing the delay length diminishes the need for high amplitude DBS signals and will reduce the side effect of stimulation currents.

**Figure 10:**
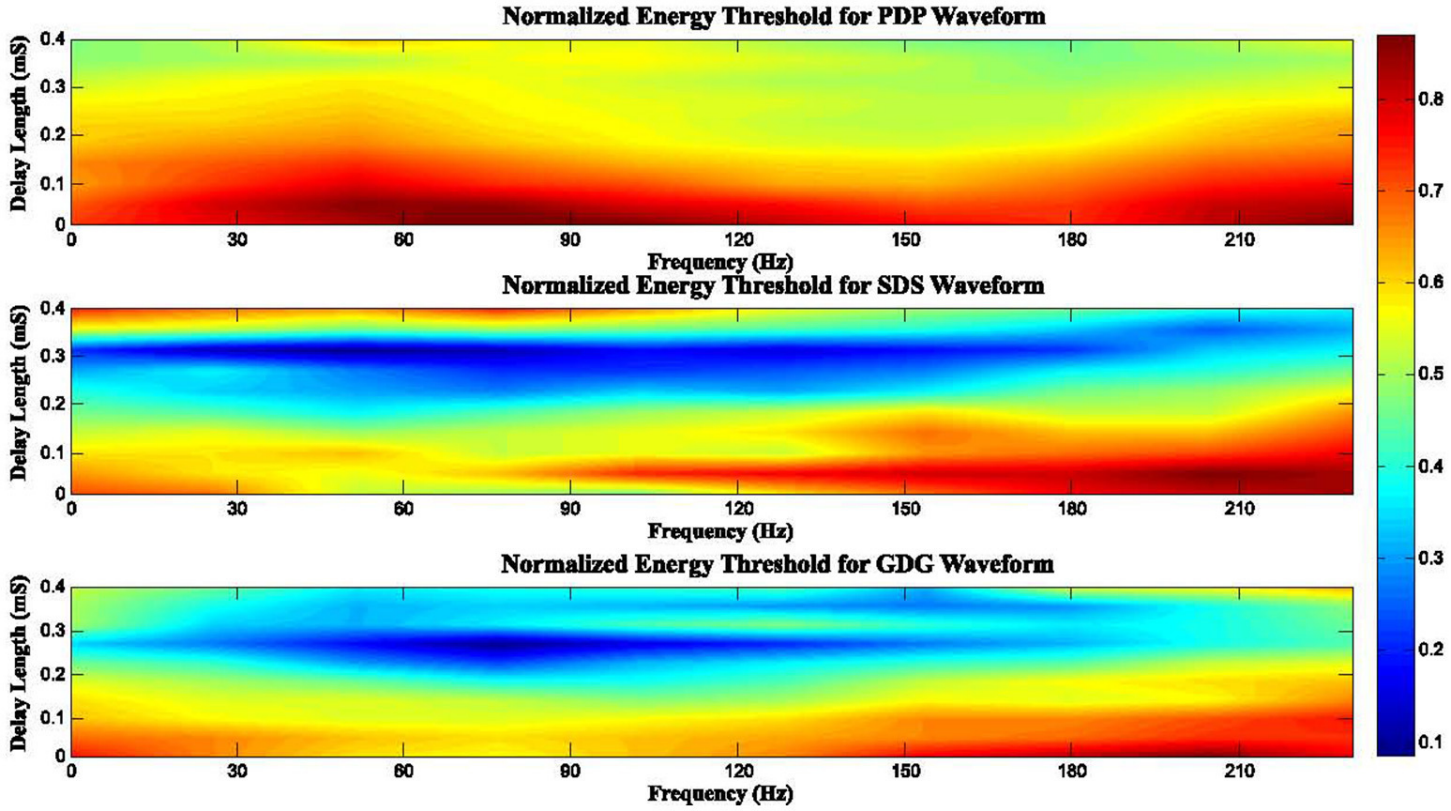
Normalized energy threshold (NET) for different DBS waveforms based on the frequency of stimulation (Fs) and delay length (DL). High frequencies cause pulses to get closer in time. In this case, the interaction between successive pulses lead to a blocking condition where the anodic phase of one pulse hyperpolarizes the membrane voltage, thus raising the threshold of the action potential for the next pulse. Therefore, an efficient DBS waveform can be determined according to a trade-off between the delay length and the frequency of DBS, as changes in frequency alters the effect of the delay.

In general, the anodic phase opposes the neural depolarization caused by the cathodic phase. Therefore, higher amplitude (more energy) is needed to evoke action potentials from neurons. Interphase delays in our model basically give enough depolarization time (reducing the need of higher DBS peaks) and also the slow uprising anodic phase of the GDG waveforms provides extra time for depolarization, making GDG the most efficient signal. Figure 10 depicts the effect of delay length and frequency of stimulation on the consumed energy. In this Figure, we defined a normalized energy threshold (NET) based on the Delay Length (DL) and Frequency of stimulation (Fs). Energy threshold was the minimum energy consumed by the DBS signal that caused at least 50% of the DBS pulses to elicit spikes. Then, we normalized the energy threshold to a range between 0 and 1. Higher values in this range indicate more energy consumed by the stimulation signal. For PDP waveforms, as long as the DL is short (DL < 0.2 mS), increase in Fs does not affect the NET (NET > 0.7). Once the DL is large enough (DL > 0.25 mS), we see a reduction of NET, especially at frequencies around 140 Hz. The DL of SDS signals showed an interesting behavior, where for 0.2 mS < DL < 0.35 mS, small NET appeared (NET < 0.3) for almost all frequencies. Fs > 120 Hz along with DL < 0.2 mS resulted in a high NET (NET > 0.8). The reason for this phenomenon is that higher frequencies indicate that pulses are getting closer in time. In this case, the interaction between successive pulses lead to a blocking condition where the anodic phase of one pulse hyperpolarizes the membrane voltage, thus raising the threshold of the action potential for the next pulse (Weitz et al., 2014). The same condition happens if the delay length is too large and successive pulses are getting close to one another. For a range of 0.2 mS < DL < 0.3 mS and 30 Hz < Fs < 140 Hz, NET was lowered (NET < 0.2) under GDG stimulation. Comparing SDS to GDG, the maximum effective DL was dropped from 0.35 to 0.3 mS, which is due to the delay-like effect of the anodic Gaussian phase, as explained before. According to the results, we conclude that there must be a trade-off between DL and the frequency of DBS, as changes in frequency alters the effect of delay. Therefore, efficient delay lengths should be selected with regards to the frequency of stimulation.

## Discussion

The results of this study show how modification of DBS waveforms in the network of the basal ganglia can be beneficial in comparison with standard rectangular DBS used in surgeries. Although, previous studies investigated the effect of a delay between the cathodic and anodic phases of DBS waveforms (Hofmann et al., 2011), examining the effect of Gaussian waveform with delay has not been fully investigated. Moreover, we implemented this signal modification on a more complex network of the basal ganglia, considering the interactions between various cell types. The computational model of the basal ganglia used in this research has some limitations which must be considered in the future. For example, neuronal firing of this model reproduced some of the experimentally observed firings and not all. For healthy and Parkinsonian's conditions, the firing rates of STN and GPi were slightly lower than the reported trends in the literature (So et al., 2012). In addition, this model does not consider the three dimensional orientation of various nuclei or the direction of neuronal projections. Although improving the model can lead to better understanding of the underlying mechanism of DBS, the model used is still able to generate the spiking rate and firing patterns for investigating DBS signal shapes. Moreover, a significant future work would be to consider the magnetic flux parameter in this neuronal population model. Neuronal firing under DBS current might be more accurate in the existence of a field of stimuli rather than current stimuli, hence it should be further investigated by the stability analysis of collective neuronal behavior (Lv et al., 2016).

The duration of the delay must be set delicately. Long delay length diminishes the need for high amplitude DBS signals and will reduce the side effect of stimulation current, but delay durations over 2 mS might not be safe for stimulation (Merrill et al., 2005). The optimal delay length can be set in a closed loop approach for each patient (Holt et al., 2016). By considering a Gaussian pulse shape for the anodic phase, we were able to reduce the delay period to 0.3 mS, which is safe to be used without tissue damaging effects of the stimulation. This is computationally studied in our work, although testing these pulse shapes experimentally is out of the scope of this paper, it is necessary to be done in future. The issue with rectangular pulses used in DBS is that their ability will decline with shorter delay lengths unless the amplitude of the pulse is also lowered relative to the reduction of the delay length. In this case, these pulses might not be effective in eliciting action potential (Cogan, 2008). DBS waveforms in Figure 8 showed that they will be able to elicit action potential with a shorter length of delay while maintaining the cathodic amplitude adequate to depolarize the membrane potential of the basal ganglia cells. The slowly uprising anodic Gaussian phase of GDG waveforms compensate for the shorter delay length. The anodic phase of the DBS waveforms also act as a hyper polarizer, causing reduction of neuronal relaxation time and therefore speeding up the bursting frequency. Faster bursting leads to more synchronization of DBS signals and neuronal firings. In addition, based on the results of Figure 9, GDG waveforms were able to achieve high PLVs with neuronal burst firing while having a shorter delay length compared to PDP and SDS specifications. In general, for all DBS waveforms in Figure 9, the proposed delay between the cathodic and anodic phases lead to higher synchronization and effectiveness. As a future direction, one can examine the effect of skewness and kurtosis of Gaussian waveforms which might have direct relation with the amount of PLV between the DBS waveform and the neuronal firings. It's been shown that GDG waveforms can achieve better results in terms of synchronization with the neuronal burst firing while having lower amplitudes. Effective low amplitude GDG waveforms decreases the energy consumption and therefore provides a longer life time of Implantable Pulse Generators (IPG), avoiding costly replacement surgeries (Hely et al., 2008).

A rectangular pulse with delay between the cathodic and anodic parts (PDP) consumed 11% less energy than a normal rectangular cathodic pulse (495 to 439 nJ) in our computational network of the basal ganglia cells with 1,000 neurons in each type. This amount of reduction was 7% for the sinusoidal pulse with delay (SDS) compared to the normal sinusoid pulse (180 to 167 nJ). For Gaussian delay Gaussian (GDG), the reduction of energy consumption of 22.5% (114 to 88.3 nJ) was achieved. Along all these waveforms, GDG was the most effective pulse shape to reduce the energy consumption. The GDG reduced the energy by 60% compared to a rectangular pulse delay pulse (PDP) waveform. In this research, we focused on the temporal specifications of the stimulus pulse as it strongly influences the performance of the treatment. More specifically, we aimed to see if modified DBS signals can reduce the amount of energy transmitted by the electrode, while being able to activate neurons within the basal ganglia region. It is crucial to consider all parameters of DBS signal specification such as amplitude, delay length, and frequency of stimulation as they all eventually play a significant role in the circuit design. Additionally, the total energy consumed by the DBS device also depends on the device pace maker circuitry, wiring and electrode specifications which must also be studied. Finally, the improvement of biophysical basal ganglia models such as considering the Autapse connections as described in Lv et al. (2016), along with various specifications of DBS signals, can provide a compelling tool for the investigation of Parkinson's disease.

## Author contributions

MD, MF, and BB came up with the ideas and discussed the analysis thoroughly. MD performed the simulations under the supervision of MF and BB. MD, MF, and BB wrote the manuscript.

### Conflict of interest statement

The authors declare that the research was conducted in the absence of any commercial or financial relationships that could be construed as a potential conflict of interest.
